# Profile of at-risk newborns attended by nurses in outpatient follow-up clinic: a retrospective cohort study*

**DOI:** 10.1590/1518-8345.2301.3113

**Published:** 2019-01-14

**Authors:** Ludmylla de Oliviera Beleza, Laiane Medeiros Ribeiro, Rayanne Augusta Parente Paula, Laíse Escalianti Del Alamo Guarda, Gessica Borges Vieira, Kassandra Silva Falcão Costa

**Affiliations:** 1 Universidade de Brasília, Faculdade de Ciências da Saúde, Brasília, DF, Brazil.; 2 Hospital Materno Infantil de Brasília, Brasília, DF, Brazil.

**Keywords:** Continuity of Patient Care, Infant, Premature, Ambulatory Care, Nursing, Follow-Up Studies, Continuidade de Assistência ao Paciente, Recém-Nascido, Prematuro, Assistência Ambulatorial, Enfermagem, Seguimentos, Continuidad de la Atención al Paciente, Recién Nascido, Prematuro, Atención Ambulatoria, Enfermería, Estudios de Seguimiento

## Abstract

**Objective:**

to analyze the cohort profile of at-risk newborns attended by nurses in a multidisciplinary follow-up clinic, with emphasis on the type of feeding and weight gain, after hospital discharge.

**Method:**

retrospective cohort, whose population is composed of at-risk newborns attended in a 4-year period. Data came from medical records and attendance report, later exported to R Program. The outcome variables were number of the nursing consultation, type of feeding, daily weight gain and main guidelines. We used descriptive statistics, frequency distribution and applied Mann-Whitney, Chi-Square, Spearman correlation, Variance and Tukey analysis, with *p* <0.05 being significant.

**Results:**

a total of 882 consultations with 629 infants and families were analyzed. The frequencies of exclusive breastfeeding and weight gain increased as the consultations progressed. The infants who needed more consultations and with lower weight gain were those with lower gestational age (p = 0.001) and birth weight (p = 0.000), longer length of hospital stay (p <0.005), and diagnoses related to extreme prematurity (p <0.05), among others.

**Conclusion:**

nurses verified the importance of outpatient follow-up of at-risk newborns, especially in promoting breastfeeding and healthy growth.

## RESUMO

**Objetivo:**

analisar o perfil de coorte dos recém-nascidos de risco atendidos por enfermeiros em Ambulatório de Seguimento Multidisciplinar, com destaque ao tipo de alimentação e ao ganho ponderal, após a alta hospitalar.

**Método:**

coorte retrospectiva, população composta por recém -nascidos de risco atendidos em período de 4 anos, dados procedentes de prontuário e relatório de atendimento, posteriormente exportados para o Programa R. As variáveis de desfecho foram: número da consulta com o enfermeiro, tipo de alimentação, ganho diário de peso e principais orientações. Houve a realização de estatística descritiva, distribuição de frequências e aplicação dos testes Mann-Whitney, Qui-Quadrado, Correlação de Spearman, Análise de Variância e Tukey, sendo significativo *p*<0,05.

**Resultados:**

foram analisados 882 atendimentos com 629 bebês e famílias. As frequências do aleitamento materno exclusivo e do ganho ponderal foram aumentando com o passar das consultas. Os bebês que necessitaram de mais consultas e com menor ganho ponderal foram os com menores idade gestacional (*p*=0,001) e peso de nascimento (*p*=0,000), maior tempo de internação (*p*<0,005) e que possuíam diagnósticos relacionados à prematuridade extrema (*p*<0,05), dentre outros.

**Conclusão:**

verificou-se a importância do acompanhamento ambulatorial de recém-nascidos de risco pelo enfermeiro, especialmente na promoção do aleitamento materno e do crescimento saudável.

## RESUMEN

**Objetivo:**

analizar el perfil de cohorte de los recién-nacidos de riesgo atendidos por enfermeros en Ambulatorio de Seguimiento Multidisciplinario, con destaque al tipo de alimentación y al aumento de peso, después del alta hospitalaria.

**Método:**

cohorte retrospectiva, población compuesta por recién-nacidos de riesgo atendidos en período de 4 años, datos procedentes de archivos e informe de atendimiento, posteriormente exportados para el Programa R. Las variables de resultado fueron: número de la consulta con el enfermero, tipo de alimentación, aumento diario de peso y principales orientaciones. Se realizó una estadística descriptiva, distribución de frecuencias y aplicación de las pruebas Mann-Whitney, Chi-Cuadrado, Correlación de Spearman, Análisis de Varianza y Tukey, siendo significativo *p*<0,05.

**Resultados:**

fueron analizados 882 atendimientos con 629 bebés y familias. Las frecuencias del amamantamiento materno exclusivo y del aumento de peso fueron aumentando con el pasar de las consultas. Los bebés que necesitaron de más consultas y con menor aumento de peso fueron los con menor edad gestacional (*p*=0,001) y peso de nacimiento (*p*=0,000), mayor tiempo de internación (*p*<0,005) y que poseían diagnósticos relacionados a la prematuridad extrema (*p*<0,05), entre otros.

**Conclusión:**

se verificó la importancia del acompañamiento ambulatorio de recién-nacidos de riesgo por el enfermero, especialmente en la promoción del amamantamiento materno y del crecimiento saludable.

## Introduction

Many are the sequelae and complications arising from the neonatal period, but the postnatal care is a critical phase, with great psychophysiological, social, economic and family changes. Improper care at this time can lead to various illnesses and death. Even so, this moment is somewhat neglected for specialized health care, which is lower in the postnatal period than before and during birth^1^.

In this scenario, we highlight the at-risk newborns (ARNB), who have the highest rates of morbidity and mortality and the highest risks of developing disabling sequelae throughout life^2^. International studies, for the most part, report that the ARNBs who receive follow-up are and/or should, basically, those premature and with low birth weight^3^ ^-^ ^4^. Other studies end up adding more risk criteria as in need for follow-up, such as small for gestational age (SGA), newborns (NB) with malformations, neonatal encephalopathy, surgical newborns, who had central nervous system infections or hyperbilirubinemia, who failed in hearing screening, who had neurobehavioral abnormalities in the neonatal period, and at-term infants who required more than 24 hours of mechanical ventilation^5^.

Regardless of the criteria used to classify the NB as at-risk or not, the surveys converge to a consensus that this population must be monitored in a differentiated, systematic and frequent way. Structured and specialized programs for the follow-up of ARNBs (especially the premature ones) are suggested and described as essential to ensure continuity of care, promote health, empower parents and families, prevent and identify early complications and illnesses, and reduce morbidity and mortality and motor, behavioral and neurodevelopmental sequelae^1^ ^-^ ^2^ ^,^ ^4^ ^-^ ^9^.

However, for this monitoring of the ARNBs to be really effective, it should be carried out by a multiprofessional and specialized team, which should be composed mainly of neonatologists, nurses, physiotherapists, occupational therapists, speech therapists, ophthalmologists, neurologists, psychologists and cardiologists^4^ ^-^ ^5^.

The role of each of these professionals in following the ARNB is evident when one observes the inherent characteristics of their professions and specialties. However, this is not a reality regarding the role of nurses working in Brazilian follow-up clinics, nor is there is any clarity regarding the profile of ARNBs served by this professional category. The literature also states that there are few national scientific productions on the outpatient follow-up of these infants by the nurse, mainly and essentially addressing premature infants^10^.

Thus, to clarify the above issues, this study was carried out with the objective of analyzing the cohort profile of at-risk newborns who were attended by nurses in a multidisciplinary follow-up clinic, with emphasis on the type of feeding and the weight gain after discharge from hospital.

Knowing this profile of care performed by nurses in the only place in the Brazilian Federal District (FD) that includes this professional category in its follow-up team makes it possible to open new paths for nursing in other local and national services, through interdisciplinarity, to ensure crucial aspects of comprehensive care for these vulnerable newborns.

## Method

This is a retrospective cohort study, and the population was composed of the ARNBs enrolled in the outpatient clinic record and attended by nurses in a reference hospital in Brasília, DF. The data collection was carried out between August and December 2016 and was referring to the consultations carried out between 2013 and 2016.

The nurse works in the follow-up outpatient clinic with nursing interns since 2010, one afternoon a week. During this period, the nurse provides care to all the ARNBs who are in the outpatient clinic for the first time (after hospital discharge) and, in subsequent consultations, in relay with the medical appointment. Babies return to see a doctor or nurse until discharge for follow-up only at the health clinic or in another day of the week (long-term follow-up). If there is any complication with the newborn, such as low weight gain, jaundice, inadequate lactation, social or psychological risk of the mother, or if the premature infant has not yet reached 3 kilos of current weight and 40 weeks of gestational age after conception, a return visit is scheduled for this baby. This visit can range from 2 days to 3 weeks (or more), according to the needs of the baby and the family. A nursing consultation is performed and, if needed, patients can be seen by a doctor, nurse, occupational therapist and speech therapist at the same day.

In the long-term follow-up, consultations are performed only by the physician and occur less frequently (preferably in developmental milestones). NBs forwarded to this follow up are those born with less than 1,500 g, less than 32 weeks of gestational age, who had Apgar less than 7 in the 5th minute, who have brain anomalies due to events in the neonatal period (periventricular leukomalacia, intraventricular hemorrhage, convulsions etc.), who had severe hypoglycemia and who are the offspring of mothers under the age of 18 years. This follow-up may occur until children turn 12 years old.

For data analysis, a dictionary and a database were built in Microsoft Excel^®^ with the study variables. The outcome variables were the number of the consultation performed by the nurse (greater number of visits, greater need thereof); type of feeding on the day of care (exclusive breastfeeding - EBF, mixed breastfeeding, use of artificial milk only, solid diet); daily weight gain; guidelines and behaviors performed by nurses. Exposure/independent variables were gestational age at birth (GA); birth weight; weight on the day of care or current weight; origin (resident in the Federal District, near the FD or outside the Federal District); number of days until the return visit, length of hospital stay; patient’s outcome (discharge to the health unit, long-term follow-up; or return visit scheduled for the same outpatient clinic); medical diagnoses of hospitalization. 

Afterwards, the data were exported to the statistical program R, version 3.3, to perform analysis of the described variables. The data were stratified according to the number of the patient’s consultation with the nurse (1st consultation, 2nd, 3rd, etc.).

Descriptive statistics (mean and standard deviation) was used for the quantitative variables, such as gestational age, birth weight, current weight, weight gain, length of hospital stay and days until the return visit. The frequency distribution was used for qualitative (or categorical) variables, such as sex, type of feeding on the day of visit, outcome, origin, diagnosis and type of nurse’s guidelines.

Subsequently, it was verified, among all the attendances, the group in which the daily weight gain was less than 20 grams, since these infants, according to the outpatient clinic’s own protocol, are considered to be at higher risk and with growth failure. Normality of the neonatal variables was tested using the Shapiro-Wilk Test. For the variables such as gestational age, birth weight, current weight, length of hospital stay and days until the return visit, the Mann-Whitney test was performed. For the variables sex, type of feeding and outcome of the patient, the Chi-Square Test was used.

To test the relationship of weight gain with the variables gestational age, birth weight and length of hospital stay, the Spearman Correlation Test was also performed. The difference between the means of weight gain and the number of the consultation/ type of feeding at the date of attendance was investigated by the Analysis of Variance (ANOVA) and the Tukey Test was used to verify what provided the difference.

This research was accepted by the Research Ethics Committee of the Teaching and Research Foundation on Health Sciences of the Federal District under the Certificate of Presentation for Ethical Assessment (CAAE) no. 55511116.6.0000.0030. Because it was a study without direct intervention in the studied population, there was no need of the Informed Consent Form (ICF) to be used). 

## Results

During the data collection period, 886 consultations were performed by nurses with 633 newborns and families. Four infants were excluded from this study because their medical records were not found (2 of 2013, 1 of 2014 and 1 of 2016). Thus, 882 consultations were performed with 629 patients (2013 = 140 visits and 104 patients; 2014 = 270 visits and 198 patients; 2015 = 215 visits and 150 patients; 2016 = 257 visits and 177 patients). It should be noted that, in 2013, there were fewer consultations by nurses due to the lack of availability of professionals.

Table 1 below shows the sociodemographic and birth profile of the babies attended by nurses, according to the number of their consultations with these nurses.

**Table 1:** Profile of at-risk newborns attended by nurses in the follow-up outpatient clinic, according to the number of the consultation to which they were submitted. Brasília, DF, Brazil, 2013-2016

Variable	1st consultation n=629 n(%)			2nd consultation n=206 n(%)		3rd consultation n=38 n(%)		4th consultation n=7 n(%)		5th consultation n=2 n(%)	
Sex											
Female	305 (48.5)			95 (46.1)		21 (55.3)		3 (42.9)		0 (0)	
Male	324 (51.5)			111 (53.9)		17 (44.7)		4 (57.1)		2 (100)	
Type of feeding											
EBF*	348 (55.3)			117 (56.8)		13 (34.2)		4 (57.14)		0	
Mixed breastfeeding	198 (31.5)			68 (33.0)		20 (52.6)		3 (42.86)		2 (100)	
Artificial milk	78 (12.4)			19 (9.2)		5 (13.2)		0		0	
Solid diet	5 (0.8)			2 (1)		0		0		0	
Outcome											
Discharged to the health unit	69 (11.0)			43 (20.9)		8 (21.1)		1 (14.3)		0	
Long-term follow-up	39 (6.2)			36 (17.5)		10 (26.3)		4 (57.1)		0	
Return visit scheduled	521 (82.8)			127 (61.6)		20 (52.6)		2 (28.6)		2 (100)	
Origin											
FD^†^	390 (62.0)			132 (64.1)		18 (47.4)		4 (57.1)		0	
Near the FD^†^	193 (30.7)			70 (34.0)		19 (50)		3 (42.9)		2 (100)	
Living outside the FD^†^	46 (7.3)			4 (1.9)		1 (2.6)		0		0	
	$\bar{X}$ ^‡^	SD^§^	$\bar{X}$ ^‡^	SD^§^	$\bar{X}$ ^‡^	SD^§^	$\bar{X}$ ^‡^	SD^§^	$\bar{X}$ ^‡^	SD^§^	
Gestational age (days)	236	24.8	233	27.3	231	23.1	214	22.8	215/	12.7	
Birth Weight (grams)	1886	647	1790	588	1678	530	1291	593	1150	523	
Current weight (grams)	2860	747	3251	778	3519	668	3591	635	3872	711	
Daily weight gain (grams/day)	29.4	16.1	36.0	14.7	35.7	25	32.6	14.7	31.5	23.3	
Length of stay	35.2	33.4	35.6	34.6	41	53.8	55.4	38.4	64	33.9	
Days until the return visit	12.2	5.8	13.9	6.4	16.3	9.1	14	0	52	53.7	

*EBF - exclusive breastfeeding; †FD - Federal District; ‡$\bar{X}$ - mean, §SD - standard deviation.

Table 1 shows that EBF is the predominant type of feeding in almost all consultations (increasing in frequency), that most of the babies (82.8%) need to return to a new visit and that there is a decrease in GA and birth weight and increase in length of hospital stay, weight gain and current weight as the consultations occur.

Added to the data in Table 1, the maximum and minimum values found, respectively, for gestational age, were 172 days (24 weeks and 5 days) and 289 days (41 weeks and 2 days), 570g and 4,100g for birth weight, 1,810 g and 7.115 g for weight at the date of the visit, -80 g/day and 170 g/day for daily weight gain, 2 and 315 days of length of hospital stay, 2 and 90 days for the number of days until the next return visit.

Of the 629 infants attended by nurses, two babies returned five times and were scheduled for yet another return visit (Table 1), which is equal to at least 10 consultations during this initial follow-up at the follow-up outpatient clinic.

**Figure 1:**
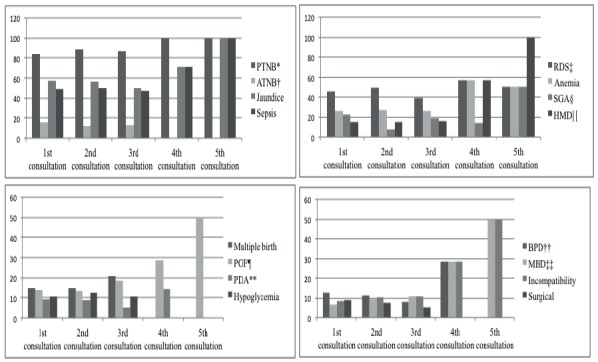
The most prevalent medical diagnoses of hospitalization in at-risk newborns seen by nurses in the follow-up clinic according to the number of the consultation to which they were submitted. Brasília, DF, Brazil, 2013-2016.

Also according to the number of the consultation/visit with the nurses, we found the most common medical diagnoses since the hospitalization of the infants, which are presented below in Figure 1.

*PTNB - preterm newborn; †FTNB - full-term newborn; ‡RDS - respiratory distress syndrome; §PIG - small for gestational age; ||HMD- hyaline membrane disease; ¶POF- patent oval foramen; **PDA - patent ductus arteriosus; ††BDP - bronchopulmonary dysplasia; ‡‡MBD - metabolic bone disease

All the diagnoses presented in Figure 1 had a frequency greater than 7.6% (n = 67) of the total of 882 visits. Other diagnoses that were not included in Figure 1 presented frequencies of 2 to 6%, some of them being in descending order: apnea, transient tachypnea of the newborn (TTNB), fungemia, enterorrhagia, malnutrition, neonatal asphyxia, genetic syndrome, intraventricular hemorrhage (IVH), convulsion, enterocolitis (ECN), conjunctivitis, cow’s milk protein allergy (CMPA), gastroesophageal reflux disease (GERD), pneumonia, retinopathy of prematurity, pulmonary hemorrhage and pulmonary hypertension. Another 94 diagnoses were identified with less frequency.

It is further verified by Figure 1 that the following medical diagnoses were present most frequently in the 4th and 5th consultations, respectively: preterm newborns (PTNB) (100%), jaundice (71 and 100%), sepsis (71 and 100%), hyaline membrane disease (HMD) (57 and 100%), respiratory distress syndrome (RDS) (57 and 50%), anemia (57 and 50%), SGA (14 and 50%), patent oval foramen (POF) (29 and 50%), Rh or ABO blood incompatibility (29 and 50%), metabolic bone disease (MBD) (14 and 50%), inguinal hernia (14 and 50%), and bronchopulmonary dysplasia (BPD) (29% and 0%).

**Figure 2:**
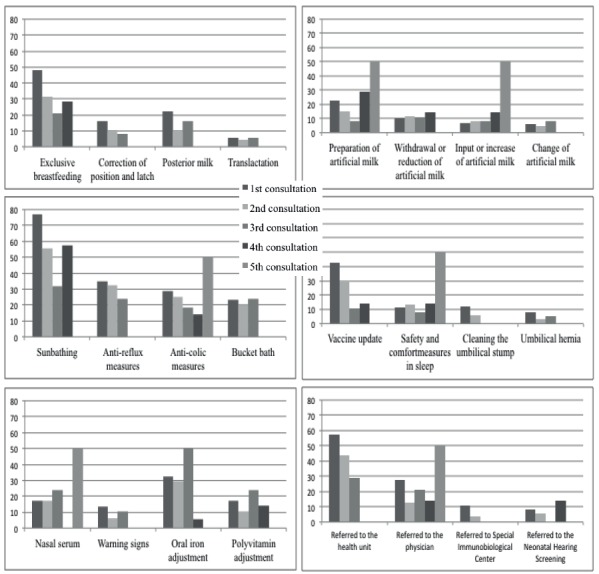
Most prevalent guidelines and behaviors in at-risk newborns attended by nurses in the follow-up outpatient clinic according to the number of the consultation to which they were submitted. Brasília, DF, Brazil.

According to the stratification by nursing consultation, additionally, the main guidelines/interventions performed were listed, which are found in Figure 2.

All of the guidelines cited in Figure 2 presented more than 7% (n = 62) of frequency during consultations and among the 76 types of guidelines and interventions listed. It was also evidenced that in the first consultation there were more than 5,000 guidelines, making an average of 8 guidelines per consultation. As new consultations occur, this average decreases to 5, 4 and 2, in the 2nd, 3rd and 4th consultations, and increases to 7 guidelines, on average, in the 5th consultation (data not included in the figure).

In the last two consultations, there were also more common guidelines/interventions, such as (Figure 2) encouragement to breastfeeding, artificial milk preparation, artificial milk intake or increase, anti-colic and sleep improvement and safety measures, nasal serum administration, developmental stimulus, adjustment of medications and referral to the doctor in the same day. 

For stratification according to the weight gain between the consultations, the Shapiro-Wilk test was performed for the variables birth weight, gestational age, weight gain and length of hospital stay. By the p-value found, we concluded that the studied population does not follow a normal distribution.

Table 2 shows the variables that demonstrate the difference in profile between the babies that obtained a weight gain lower than and greater than 20 g/day.

**Table 2:** Profile of at-risk newborns attended by nurses in the follow-up outpatient clinic according to the degree of weight gain and the significance of the statistical tests performed. Brasília, DF, Brazil, 2013-2016

Variable		Weight gain < 20g/day n=190		Weight gain > 20g/day n=692		p*
		$\bar{X}$ ^†^	SD^‡^	$\bar{X}$ ^†^	SD^‡^	
Gestational age (days)		232.2	27.5	235.6	24.3	0.001^§|^
Birth weight (grams)		1785.8	689.6	1866.1	615.8	0.000^§^
Current weight (grams)		2769.9	816.6	3048.2	757.3	0.000^||^
Length of hospital stay (days)		44.4	42.5	33.4	32.1	0.000^§^ 0.003^||^
Days until the return visit		9.1	7.5	14.0	6.0	0.000^||^
		f^¶^	%	f^¶^	%	p*
Sex	Female	96	50.5	328	47.4	0.002^||^
	Male	94	49.5	364	52.6	
Type of feeding	- EBF**	102	53.7	380	54.9	0.060^††^ 0.012^‡‡^
	- Mixed breastfeeding	57	30.0	234	33.8	
	- Artificial milk	27	14.2	75	10.8	
	- Solid diet	4	2.1	3	0.5	
Outcome	- Discharge for the health unit	8	4.2	113	16.3	0.000^††^
	- Long-term follow-up	9	4.7	80	11.6	
	- Return visit scheduled	173	91.1	499	72.1	0.000^††^

*p - α=0.05; †$\bar{X}$ - mean; ‡SD - standard deviation; §Spearman Correlation Test Results; ||Mann-Whitney Test Results; ¶f - frequence; **EBF - exclusive breastfeeding; ††Chi-Square Test Results; ‡‡Results of Analysis of Variance (ANOVA), but this result was not confirmed by the Tukey Test

Table 2 shows that the Spearman Correlation test evidenced a significant correlation between weight gain and the variables GA, birth weight and length of hospital stay, with the following additional values of correlation and p-values, respectively: 0.110/0.001, 0.127/0.000 and -0.114/0.000. 

The application of the Chi-square test between the low weight gain and the variables shown in Table 2 related to the patient’s outcome showed that there is a significant association between discharge from the outpatient clinic and whether the baby should return, that is, the fact that the baby has had a low weight gain influences whether he/she will be discharged from the outpatient clinic or if he/she will have a scheduled return visit.

When the Mann-Whitney test was applied, there was a significant difference between the variables current weight, number of days until the return visit and length of hospital stay with the fact that the baby was classified or not as with low weight gain (Table 2). Through this same test, it was possible to verify that values of weight gain of less than or equal to 20g/day have a significant relation with the following medical diagnoses: hypoglycemia (p = 0.040), BPD (p = 0.026), apnea of prematurity (p = 0.001), ventricular/biventricular dilatation (p = 0,000), MBD (p = 0.004), pulmonary hemorrhage (p = 0.006), pulmonary hypertension (p = 0.003), atelectasis (p = 0.002), congenital heart diseases (p = 0.043), genetic syndromes (p = 0.044), anemia (p = 0.045) and diaphragmatic eventration (p = 0.001) (data not included in the table).

The analysis of variance (ANOVA) was used to verify whether there is a difference between the weight gain and the feeding groups (EBF, mixed breastfeeding, artificial feeding and solid diet), origin (FD-cities, cities of Goiás and Minas Gerais). By the p-value, a significant difference of weight gain was found according to the type of feeding (p = 0.012), but not for origin (p = 0.616) (Table 2). In order to verify which type of feeding provided this difference, we applied the Tukey’s test between each type (mixed breastfeeding-EBF: p = 0.568, artificial breastfeeding-EBF: p = 0.34, solid diet-EBF: p = 0.119, mixed breastfeeding-artificial feeding: p = 0.075, mixed breastfeeding-solid diet: p = 0.068, solid diet-artificial feeding: p = 0.328). As the p-value was greater than 0.05 in all possible comparisons, we concluded that there was no influence of the type of feeding in the weight gain of the babies.

When comparing the weight gain between the consultations also with the ANOVA, it was noticed that there is a significant difference of weight gain in at least one of the 5 visits (p = 0.000). The Tukey’s test was then performed, comparing the two-to-two consultations, finding a significant difference in weight gain only between the 1st and 2nd consultation (p = 0.000, difference 6.58, confidence interval 3.01 -10.16) (data not included in the table). 

It should be added to these data that the guidelines/interventions that had a statistical difference and were more frequent in low weight gain babies were encouragement to breastfeeding (p = 0.025), correction of position and latch (p = 0.000); posterior milk (p = 0.000); translactation (p = 0.000); artificial milk preparation (p = 0.004); intake or increase of artificial milk (p = 0.000); referral to physiotherapy (p = 0.032) and speech therapy (p = 0.007).

## Discussion

This was the only national study that screened and analyzed the profile of ARNBs attended by nurses in a follow-up clinic in the Federal District, presenting the largest number of participants and attendances performed by this professional category. It has also been demonstrated that the importance of nursing care is not only found in the quantitative evolution of care, but also in the extent to which they are able to improve EBF and patient’s growth. There were also factors influencing the weight gain of ARNBs and the main guidelines/interventions performed with those who gained little weight. 

In other studies, nurses also had crucial roles in ARNBs follow-up, from its planning and operationalization, education, guidance and parent training^11^ ^-^ ^15^, referrals to other specialties^5^ ^,^ ^12^, home visit^11^ ^-^ ^12^ ^,^ ^15^ ^-^ ^17^ ^)^ and telephone availability for questions^11^ ^-^ ^12^ ^,^ ^16^ up to family support, assessment/maintenance of babies’ health and well-being^11^ ^,^ ^15^ ^-^ ^16^ ^,^ ^18^ ^-^ ^19^ and support to the EBF^12^ ^-^ ^13^. The results also led to improved quality of care, increasing parental confidence^18^, reducing length of hospital stay^11^ ^,^ ^16^, improving weight gain^13^ ^,^ ^17^, the rate of immunizations and maternal satisfaction, reducing costs and readmissions^9^ ^,^ ^16^.

Considering that described in Table 1, it was possible to notice that the sex of the NB attended was relatively balanced. Only in the last consultations, the male gender was predominant, but in relation to the weight gain, the female gender found statistical significance. Studies that indicate the profile of the attendees also vary in the predominance of males^17^ or females^10^ ^,^ ^20^ ^-^ ^21^, and other studies related to growth also show that girls are the ones that gain less weight during outpatient follow-up^21^ ^-^ ^22^.

The first visit to the follow-up clinic under study is around the 3rd to 9th day after discharge. A retrospective American study that evaluated 65,085 discharges demonstrated that less than seven days after discharge is the ideal time for this first consultation to occur (for reducing readmissions)^23^. The average of EBF in this first consultation was around 55% and of breastfeeding in general was above 85%. This frequency of EBF can be considered adequate if compared to that found in an Israeli study that showed that only 109 of 162 mothers (67%) were breastfeeding (exclusively or not) their preterm infants at the time of hospital discharge^24^. However, if confronted with a prospective cohort of 137 preterm infants performed in the northeast region of Brazil, the frequency is below the 56.2%, found on discharge from the Kangaroo ward^20^, being necessary to consider that the Kangaroo Method was related to increased EBF in other researches^12^ ^,^ ^25^. It should be noted that, as in this study, in another publication from Paraná state, a descriptive and retrospective study of 25 premature infants, it was shown that the growth was not altered according to what the baby was ingesting^10^.

Some findings of this research allow us to infer that the follow-up clinic has promoted and stimulated EBF. For example, there was an increase in the frequency thereof from the first to the second consultation and from the second to the fifth consultation, and the withdrawal and/or reduction of the artificial milk was greater than the input and/or increase thereof among the guidance provided.

Data on patient’s outcome presupposes that as 11% of the ARNBs often have only one consultation with the nurse and are already referred to the Health Unit or Primary Care Unit; they are adequately growing and developing or are referred to service networks (such as Genetics and Congenital Infections). And, since less than half of the 521 babies with a scheduled return visit came back to a second consultation, the outpatient clinic’s resolution could be verified just as the importance of the first consultation with the nurse.

Regarding the origin, about 40% of the babies attended in the Ambulatory are from neighboring states, such as Goiás and Minas Gerais. This fact may lead to greater dropout of this follow-up, as this is, sometimes, related to the greater distance between the residence and the follow-up clinic^12^ ^,^ ^26^.

Regarding the data on GA and birth weight present in Table 1, this study found that the lower the gestational age and the birth weight, the more frequent and the greater the follow-up visits are. Some researches show this need for more frequent follow-up when they affirm that low birth weight and prematurity are related to an increase in morbidity and mortality, chronic conditions and readmissions^2^ ^-^ ^3^ ^,^ ^9^ ^,^ ^19^ ^,^ ^27^ ^-^ ^31^, as well as to the greater risk of growth deficit^3^ ^,^ ^21^, developmental delays and cognitive and behavioral problems^3^ ^,^ ^7^ ^,^ ^31^ ^-^ ^32^. In addition, growth must be closely monitored in the first year of life of patients with this profile, thus ensuring optimal brain nutrition and reducing risks of neurodevelopmental delays^19^ ^,^ ^33^.

It was also observed that the longer the infant’s length of hospital stay, the greater the need for return visits and the greater risk of lower weight gain. Since length of hospital stay directly correlates with GA^3^, this data also corroborates a study carried out in Sweden with 1,410 premature infants on an early discharge program, in which the ones that had more readmissions were those with longer hospital stay^11^, that is, greater length of hospital stay is related to higher risk of morbidities and growth deficit.

The ascending evolution of the current weight over the consultations, just like that of the weight gain until the third consultation, shows the effectiveness of the follow-up clinic in guaranteeing an adequate ARNB growth, which was also evidenced in another research^10^.

It is even known that weight gain is an important diagnoser of baby’s health^11^, and a statistically significant difference could be verified from the first to the second consultation regarding weight gain in this study. This may indicate that the assistance provided to the ARNBs was effective and that the guidance provided at the initial consultations had a positive impact on growth.

Regarding the most common medical diagnoses of hospitalization present in the served population, these were equivalent to others found in follow-ups performed by nurses^10^. Especially with regard to the last consultations, it is believed that the presence of these diagnoses may even be considered as a risk factor for postnatal complications, and thus they should be closely monitored.

Most of these diagnoses are found in premature infants born with less than 1,500g, neuropathic and those with longer length of stay^2^ ^-^ ^3^ ^,^ ^19^, and in some studies, they are also present in chronic infants coming from the NICU^27^ and in those who most needed readmissions^9^ ^,^ ^11^ ^,^ ^29^. Neonatal conditions, such as prematurity, asphyxia, infections and hyperbilirubinemia, were associated with sequelae and compromised survival in a retrospective Italian cohort performed with 123 subjects^7^. Anemia must be monitored and treated frequently because it can lead to low weight gain and changes in development^19^ ^,^ ^33^. Incompatibility, jaundice, malnutrition and enterorrhagia had a greater need for return visits in order to verify and guarantee the reversibility of these problems.

Some medical diagnoses were also found to be statistically significant for weight gain, in addition to prematurity and low birth weight, namely BPD, pulmonary hemorrhage and hypertension, MBD, apnea of prematurity, ventricular dilatation, anemia, pneumothorax, atelectasis, hypoglycemia, congenital heart diseases, diaphragmatic eventration and genetic syndromes, being associated, in other studies, with various morbidities.

Premature infants with chronic lung disease may have a delay in oral feeding proficiency^14^ ^,^ ^19^. In a descriptive study with preterm infants in follow-up, birth diagnoses referring to extreme prematurity (such as the first seven diagnoses mentioned above) were associated with weight loss^10^. An international protocol for the care of late preterm infants revealed that patients with respiratory and cardiac disorders are more susceptible to feeding problems and consequently to failure of growth^28^. A prospective cohort conducted in Paraná with 237 ARNBs confirmed that low birth weight associated with prematurity, mother under 17 years old and congenital anomalies were risk factors added to readmissions (which are even longer than infants without these aspects) until the third month of life^9^. A disease can obviously alter growth, since the weight gain is lower during a illness^10^. No association was found in the literature regarding the diagnosis of hypoglycemia, pneumothorax, atelectasis and diaphragmatic eventration with failure of growth.

The most common and most frequent guidelines to ARNBs were those related to infant feeding, corroborating other studies that show that the major problems and needs of supervision are those of nutrition, besides growth failure, respiratory morbidities, anemia and neurodevelopmental sequels^4^ ^,^ ^14^ ^,^ ^28^ ^,^ ^33^. Some studies already indicate that the main guidelines were similar to those found in this one, such as vaccination, medications and hygiene^12^. Studies also point to specific postnatal care that could improve neonatal health and were identified as being present at the follow-up care of the study hospital, such as support for EBF, body hygiene and umbilical stump cleanup^34^ ^-^ ^35^.

The referral to the doctor, performed by nurses on the same day of care, was about 25%. The reasons for this referral varied, but were based on the need to evaluate patients with complications and with the need for medication prescriptions and requests for tests. Thus, the family would not need wait for complication of the clinical picture or for a next consultation to resolve these problems. This ratifies that the ARNB monitoring by a multidisciplinary team is much more appropriate and necessary for them and their families from the physical, socioeconomic and emotional point of view^28^.

This multidisciplinary understanding of care is confirmed by the number of referrals performed by the nurse and by the relay between nursing and medical consultations. This relay has already been advocated in an exploratory and qualitative research from Santa Catarina that interviewed 31 health professionals of Primary Care^12^ and may lead to decreased readmissions during the first year of life^4^ and to ensuring appropriate care for ARNBs, especially premature infants^19^.

Since the first consultation with the nurse, the bond of the parents and other family members with the baby is confirmed or refuted, and the bonds of these with the professional are strengthened. This fact and joint accountability are essential for successful follow-up^12^ ^,^ ^18^. And as the doctor and the nurses who work in the outpatient clinic work in other neonatal units of the hospital, this strengthening is facilitated. Parents are more comfortable if the professional who attends them after discharge works in another sector of the hospital^15^ and tend to rely more on NICU nurses to provide guidance at this level of care^18^.

The results of this study confirm that the major risk factors related to the morbidity and mortality of ARNBs could be minimized through actions of health promotion and disease prevention^2^. Some papers advocate the integration of the Outpatient Follow up with Primary Care as a strategy to ensure continuity of care to the ARNB, systematization of care, professional training, implementation of programmatic actions and protocols, and with the due monitoring of results^2^ ^,^ ^12^ ^,^ ^14^ ^,^ ^36^. 

The limitations of this research were those related to the use of secondary data of retrospective data collection, which can not only influence the quality of these but also make it impossible to deepen them. Thus, social variables, such as the mother’s income, occupation and schooling, could not be rescued, preventing a greater enrichment of this research. However, it is expected that what has been explained here may leverage the interest of new and necessary research in the area, especially prospective ones and those that explore, in this multiprofessional context, the nursing diagnoses and interventions, as well as their corresponding outcomes.

## Conclusion

This study allowed the analysis of the profile of a large number of ARNBs attended by nurses in an outpatient follow-up clinic of a reference hospital for maternal and child health in the Brazilian FD, over a four-year period. It was verified that the population served by nurses is mostly composed of newborns with low weight and gestational age below 34 weeks at the time of birth, in exclusive breastfeeding, with length of hospital stay greater than 30 days, who needed more than one monthly consultation at the outpatient clinic, which had problems to gain weight and medical diagnoses that may lead to failure in growth and development.

This profile analysis provided the knowledge that it is possible to perform nursing consultation with this population, with well-defined roles, and that this care can improve both exclusive breastfeeding and weight gain. These factors, among others, make it possible to provide qualified and continuous assistance to such vulnerable babies. Knowledge of the ARNB profile provided by this study also allows that nurses explore other follow-up models, thus favoring comparisons with populations in different locations, and study of other possible risk factors for a healthy growth and development of ARNBs.

This research also demonstrated how an outpatient follow-up should be structured, based on multiprofessional care. Everyone can work within their training area, but when the various professionals join forces and knowledge, the biggest beneficiaries are the infants and their families.

In order to further improve nursing care for ARNBs, the integration of the outpatient follow-up service with Primary Care should be consolidated, preferably in the form of a home visit shared by nurses, at least during the first year of life of these babies, when the risks and vulnerabilities to morbidities are greater. The nurses would be linked to both the neonatal hospital units and the Family Health Program, reinforcing their role and collaborating with the necessary reference and counter-reference services. 

## References

[1] World Health Organization (2013). Recommendations on postnatal care of the mother and newborn.

[2] Faria CS, Martins CBG, Lima FCA, Gaíva MAM (2014). Morbidity and mortality among the high-risk newborns: a
bibliography review. Enferm Global.

[3] Glass HC, Costarino AT, Stayer SA, Brett CM, Cladis F, Davis PJ (2015). Outcomes for extremely prematures infants. Anesthesia Analgesia.

[4] Bockli K, Andrews B, Pellerite M, Meadow W (2014). Trends and challenges in United States neonatal intensive care
units follow-up clinics. J Perinatol.

[5] Doyle LW, Anderson PJ, Battin M, Bowen JR, Brown N, Callanan C (2014). Long term follow up of high risk children: who, why and how?.
BMC. Pediatrics.

[6] World Health Organization (2015). Health In 2015: From MDGs TO SDGs.

[7] Poggioli M, Minichilli F, Bononi T, Meghi P, Andre P, Crecchi A (2016). Effects of a home-based family-centered early habilitation
program on neurobehavioural outcomes of very preterm born infants a
retrospective cohort study. Neural Plast.

[8] Santos HG, Andrade SM, Silva AMR, Mathias TAF, Ferrari LL, Mesas AE (2014). Avoidable causes of infant deaths due to interventions of the
Brazilian Unified Health System a comparison of two birth
cohorts. Ciênc Saúde Coletiva.

[9] Barreto MS, Silva RLDT, Marcon SS (2013). Morbidity in children of less than one year of age in risky
conditions: a prospective study. Online Braz J Nurs.

[10] Viera CS, Rech R, Oliveira BRG, Maraschin MS (2013). Preterm infant follow up during the first year after hospital
discharge: assessing weight-height development. Rev Eletr Enferm.

[11] Lundberg B, Lindgren C, Palme-Kilander C, Örtenstrand A, Bonamy AKE, Sarman I (2016). Hospital-assisted home care after early discharge from a Swedish
neonatal intensive care unit was safe and readmissions were
rare. Acta Paediatr.

[12] Aires LCP, Santos EKA, Costa R, Borck M, Custódio ZAO (2015). Baby follow-up in primary care interface with the third stage of
the kangaroo method. Rev Gaúcha Enferm.

[13] Dehkhoda N, Valizadeh S, Jodeiry B, Hosseini MB (2013). The effects of an educational and supportive relactation program
on weight gain of preterm infants. J Caring Sci.

[14] Lipner HS, Huron RF (2018). Developmental and interprofessional care of the preterm infant:
neonatal intensive care unit through high-risk infant
follow-up. Pediatrics Clin N Am.

[15] Lopez G, Anderson KH, Feutchinger J (2012). Transition of premature infants from hospital to home
life. Neonatal Network.

[16] Bryant-Lukosius D, Carter N, Reid K, Donald F, Martin-Misener R, Kilpatrick K (2015). The clinical effectiveness and cost-effectiveness of clinical
nurse specialist-led hospital to home transitional care a systematic
review. J Eval Clin Pract.

[17] Miró RA, Canut ML, Aloy JF, Ruiz ME, Gili LA, Rodríguez JB (2014). Influence of in-home nursing care on the weight of the early
discharged preterm newborn. Anales de Pediatría (Barc).

[18] Adama EA, Bayes S, Sundin D (2016). Parents' experiences of caring for preterm infants after
discharge from Neonatal Intensive Care Unit A meta-synthesis of the
literature. J Neonat Nurs.

[19] Barkemeyer BM (2015). Discharge planning. Pediatr Clin N Am.

[20] Menezes MAS, Garcia DC, Melo EV, Cipolotti R (2014). Preterm newborns at Kangaroo Mother Care a cohort follow-up from
birth to six months. Rev Paul Pediatr.

[21] Sammy DM, Chege MN, Oyieke J (2016). Early growth in preterm infants after hospital discharge in rural
Kenya longitudinal study. Pan African Med J.

[22] Kattula D, Sarkar R, Sivarathinaswamy P, Velusamy V, Venugopal S, Naumova EN (2014). The first 1000 days of life prenatal and postnatal risk factors
for morbidity and growth in a birth cohort in southern India. BMJ Open.

[23] Jackson C, Shahsabebi M, Wedlake T, DuBard CA (2015). Timeliness of outpatient follow-up an evidence-based approach for
planning after hospital discharge. Ann Fam Med.

[24] Pinchevski-Kadir S, Shust-Barequet S, Zajicek M, Leibovich M, Strauss T, Leibovich L (2017). Direct feeding at the breast is associated with breast milk
feeding duration among preterm infants. Nutrients.

[25] Briere CE, McGrath J, Cong X, Cusson R (2014). An integrative review of factors that influence breastfeeding
duration for premature infants after NICU hospitalization. J Obstet Gynecol Neonat Nurse.

[26] Ballantyne M, Stevens B, Guttmann A, Willan AR, Rosenbaum P (2013). Maternal and infant predictors of attendance at neonatal
follow-up programmes. Child: Care, Health Develop.

[27] Tavares TS, Duarte ED, Silva BCN, Paula CM, Queiroz MPM, Sena RR (2014). Profile characterization of children discharged from neonatal
units presenting chronic conditions. Rev Enferm Centro Oeste Mineiro.

[28] Phillips RM, Goldstein M, Hougland K, Nandyal R, Pizzica A, Santa-Donato A (2013). Multidisciplinary guidelines for the care of late preterm
infants. J Perinatol.

[29] Martins CBG, Barcelon AA, Lima FCA, Gaíva MAM (2014). Profile of morbidity and mortality in at-risk
newborns. Cogitare Enferm.

[30] Lansky S, Friche AAL, Silva AAM, Campos D, Bittencourt SDA, Carvalho ML (2014). Birth in Brazil survey: neonatal mortality profile, and maternal
and child care. Cad Saúde Pública.

[31] Milner KM, Duke T, Steer AC, Kado JH, Koyamaibole L, Kaarira R (2017). Neurodevelopmental outcomes for high-risk neonates in a
low-resource setting. Arch Dis Childhood.

[32] Pierrat V, Marchand-Martin L, Arnaud C, Kaminski M, Resche-Rigon M, Lebeaux C (2011). Neurodevelopmental outcome at 2 years for preterm children born
at 22 to 34 weeks' gestation in. France in.

[33] Kirk CM, Uwamungu JC, Wilson K, Hedt-Gauthier BL, Tapela N, Niyigena P (2017). Health, nutrition, and development of children born preterm and
low birth weight in rural Rwanda a cross-sectional study. BioMed Central Pediatrics.

[34] Salam RA, Mansoor T, Mallick D, Lassi ZS, Das JK, Bhutta ZA (2014). Essential childbirth and postnatal interventions for improved
maternal and neonatal health. Reproductive Health.

[35] Lee ACC, Kozuki N, Cousens S, Stevens GA, Blencowe H, Silveira MF (2017). Estimates of burden and consequences of infants born small for
gestational age in low and middle income countries with INTERGROWTH-21st
standard analysis of CHERG datasets. Br Med J.

[36] Aires LCP, Santos EKA, Bruggemann OM, Backes MTS, Costa R (2017). Reference and counter-reference health care system of infant
discharged from neonatal unit perceptions of primary care health
professionals. Esc Anna Nery.

